# Investigating the Influence of Ellipticity on the Purcell and Quality Factors in Planar–Elliptical Bragg Mirrors

**DOI:** 10.1155/tswj/1033773

**Published:** 2025-05-01

**Authors:** Sanaa Al-Sumaidae

**Affiliations:** Automated Manufacturing Engineering Department, Al-Khwarizmi College of Engineering, University of Baghdad, Baghdad, Iraq

**Keywords:** elliptical microcavity, ellipticity, Purcell factor

## Abstract

High-quality factor *Q* and elliptical cross-section microcavity of small size are described. We present a numerical investigation of the performance of the elliptical microcavity. We design elliptical microcavities to control the emission rate of dipolar emitters and investigate how the ellipticity factor influences the Purcell and *Q*-factors. We demonstrate an enhancement of up to 16 × 10^3^ in the Purcell factor for TiO_2_-based mirrors and 8 × 10^3^ for ZnS-based mirrors. A numerical study at 1550 nm also shows that an ellipticity factor (*ε* = 0.4) could significantly affect the Purcell and *Q*-factor. These benefits are expected to be even more persuasive in short wavelengths.

## 1. Introduction

Optical microcavities are strong platforms commonly used in the fields of quantum information and integrated photonic [1]. Cavity QED [2–4], nonlinear optics [5], and vertical-cavity surface-emitting lasers [6, 7] are only a few of the applications for microcavities. Enhancing the interaction between light and matter is made possible by optical microcavities, which trap photons in a small spatial volume with long lifetimes [1]. In the domain of cavity quantum electrodynamics (cQED), these microcavities provide a wide range of applications, including the development of quantum light sources [8], the modification of spontaneous emission [9], and the construction of microlasers [10].

Optical resonators can be functional in achieving strong coupling between exciton resonance and photons of the cavity [11]. A common microcavity design like a Fabry–Pérot resonator encompasses two distributed Bragg reflectors. This microcavity can be assembled from a monolithic structure or multiple independent parts [12–14] . Alternatively, an open-cavity approach involves assembling it from two independent mirrors with a controllable distance between them [15]. These cavities can employ different types of mirrors, including dielectric [16], semiconductor [17], or metallic [18].

Recently, the cavity QED research community has shown a great deal of interest in the “open cavity” (i.e., where the gap consists of air or vacuum), the hemispherical mirror Fabry–Pérot cavity [19–23]. One of the main benefits of this configuration is the capacity to adjust the resonant frequency of the modes by altering the distance between mirrors, as well as the capability to place emitters and field antinodes within the “empty” region between mirrors. High Purcell factor (*F*_*P*_) and quality factor are desired for many applications of interest, which drives the need to build cavities with small mode volume (*V*_*m*_) and high-quality factor (*Q*). Since the strength of the coupling between emitters and photons is inversely proportional to the mode volume, a small mode volume is highly suitable in cQED applications. However, because the quality factor (*Q*-factor) degrades, the diffraction limit prevents microcavities from becoming smaller on the wavelength scale [24]. The most optimal light–matter interaction strength must therefore be obtained by striking a balance between *V*_*m*_ and *Q*-factor. Researchers have investigated several optical modes in various microcavity systems in an effort to find the optimal *Q*/*V*_*m*_ ratio. In microdisks and microtoroid cavities, whispering gallery modes have a huge mode volume but an exceptionally high *Q*-factor (> 10^8^) [25]. Conversely, defect modes in photonic crystals have a balanced *Q*-factor and mode volume [26], whereas surface plasmon modes in metal nanostructures have a tiny mode volume (*V*_*m*_ ≪ *λ*^3^, where *λ* is the vacuum wavelength) but are unable to reach a high *Q*-factor [27].

The quantity of electromagnetic states that a photon can occupy at a certain location inside a system is measured by the local density of optical states (LDOS). There, light–matter interaction will be improved by a high localization of the LDOS. The Purcell factor, *F*_*P*_, which was initially responsible for the augmentation of an atom's spontaneous emission in a cavity [28], is obtained from the LDOS at resonance. Due to the little absorption loss, dielectric cavities can have high *Q*-factor values, which can significantly increase the Purcell factor [29]. We previously demonstrated [30] that the omnidirectional characteristic offers a great deal of potential for separating emitters from radiation modes in free space. Another theoretical study for dome cavity [31] investigates the application of amorphous hydrogenated silicon as a high-refractive-index material in QWS Bragg mirrors specifically designed for applications in cQED. The study reveals significant improvements in high Purcell factor, cooperativity, and spontaneous emission coupling factor. The cavity utilized in this research is a buckled-microcavity Fabry–Pérot resonator. We carried out a prior study examining the optical characteristics of these devices, which includes their capability for fabrication with significant birefringence and nondegeneracy in polarization mode [32]. Moreover, this cavity enables the integration of numerous materials inside the cavity. Material systems, where photons can excite resonance cavity mode through dipole transitions, are especially interesting. However, the investigation of the Purcell factor and *Q*-factor for these types of microcavity has not been reported yet. This paper investigates ellipsoid variants of buckled-dome monolithic microcavities, which have been utilized to establish a monolithic method that employs a controlled thin film buckling process [33, 34]. An initial attempt to incorporate optical mechanical resonators [35] and liquid infiltration [36] into this method has previously been published. Here, we investigated how microcavity shape affects the Purcell factor while taking ellipticity and size into account. We conducted detailed finite-difference time-domain (FDTD) simulations on elliptical optical structures to elucidate the resonance cavity interaction between emitters and fields for the generation of the Purcell factor.

## 2. The Structure of Elliptical Microcavity Modelling

**Figure 1:**
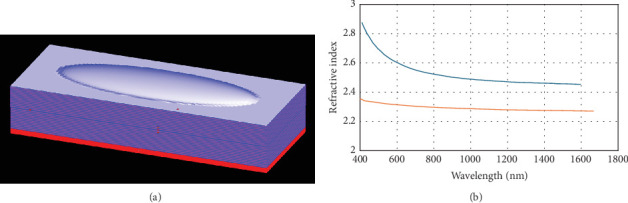
(a) Perspective view for elliptical dome microcavity in cross-section. The top (dome ellipsoid) and lower (planar) mirrors are thought to be the same *N*(1/2)-period QWS. (b) The refractive index in the form of a function of wavelength is considered for TiO_2_ (represented by blue solid curves) and ZnS (depicted by red curves) films in our modelling case.

For the purpose of studying the modelling, we stimulate different geometries and materials of plano elliptical cavities, labelled as “ZnS-based” and “TiO_2_-based” ellipsoid microcavities. These cavities exhibit variation in their ratios of the major to minor axes. The cavity structure used here consists of two mirrors, which is demonstrated in Figure 1. We consider *N*(1/2) period of quarter-wave-stack (QWS) mirrors, composed of alternating layers with high- and low-refractive indices in the quarter-wave thickness configuration. The low-index layers in both scenarios are assumed to be SiO_2_. High-quality SiO_2_ films generally show very low absorption losses; hence, we employ a standard (lossless) dispersion model for the SiO_2_ films (*n* ~ 1.45 at 1550 nm) [34]. A cross-sectional perspective picture of an elliptical dome microcavity assumes that the lower (planar) and upper (dome ellipsoid) mirrors are the same *N*(1/2) -period QWS. Modelled ellipsoid mirrors face outward like a dome, as seen in Figure 1.

It is worth noting that the interface between the upper dome and the QWS mirror is deliberately curved, not flat. The dome structure is designed as an elliptical surface, in which the curvature is essential because it directly improves the microcavities' optical performance, showing that the dome's function goes beyond just acting as a substrate.

Figure 1 gives refractive indices of zinc sulfide (ZnS) and titanium dioxide (TiO_2_), respectively, at 1550 nm wavelength. Quarter-wave layer thicknesses of 265 nm for SiO_2_, 158 nm for TiO_2_, and 170 nm for ZnS are used, respectively. We assume that light enters from the air (*n*_air_ ~ 1) and exits through silica (substrate) medium (*n*_*s*_ ~ 1.46) unless otherwise specified. Keep in mind that the primary conclusions are not significantly affected by the exit medium selection.

**Figure 2:**
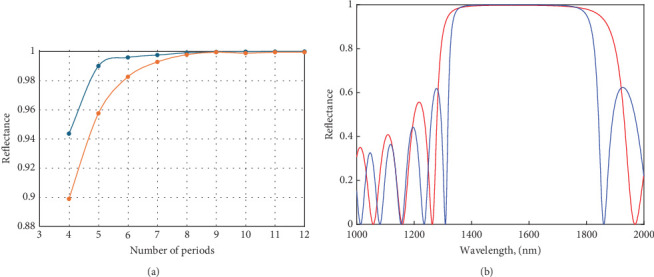
(a) Estimated reflectance at a 1550 nm wavelength, depending on the number of period present in a quarter-wave-stack (QWS) mirror containing high-index layers of ZnS (red circle) and TiO_2_ (blue circles). (b) Reflectance predictions at normal incidence for 13(1/2)-period TiO_2_-based (red line) and ZnS-based (blue line) QWS mirrors.

To study these trade-offs extensively, we used transfer–matrix methods. For both mirrors under consideration, the reflectance and transmission at wavelength 1550 nm are plotted versus the period count in Figure 2a. To achieve the maximum reflectivity for mirror-based ZnS maximum, *R*_max_ requires 13(1/2) layers, while mirror-based TiO_2_ requires fewer layers to achieve a maximum reflectance *R*_max_ of around 7(1/2). However, in the simulation, we did both mirror 13(1/2) layers to get high reflectance.

The normal–incidence reflectance for each mirror is plotted, as illustrated in Figure 2b. Note that the stop band in the TiO_2_-based mirror is a little bit wider than the ZnS-based mirror, due to its significantly higher-index contrast. Based on transfer–matrix simulations, the reflectance of the top and bottom mirrors is estimated to be RTM ~ 0.990 and RBM ~ 0.985, respectively. This affects how spontaneous emission is controlled, as will be covered in more depth later. We employ the FDTD method to determine the optical characteristics of the elliptical microcavity, such as cavity spectra, optical field distributions, mode volumes, and *Q*-factors.

## 3. Spontaneous Emission in ZnS and TiO_2_ Elliptical Microcavities (Comparison of the Distinction Between the Two Categories of Structures)

To enable a numerical analysis of cavity impacts, a cavity model was employed that was fabricated in our previous research [32]. The model system is an elliptical microcavity comprised of alternating multilayers of Material A (TiO_2_(ZnS)) and air and Material B (SiO_2_) with refractive index (2.45(2.25)) and 1.45, respectively, at 1550 nm modelled in the form (AB)^*N*^air(AB)^*N*^. Here, we suppose that there are two distinct devices of elliptical cavities, which we will represent “ZnS-based” and “TiO_2_-based” ellipsoid cavities. These cavities differ slightly regarding their major and minor axis ratios. In this case, the cavity length was considered to be *d*_*A*_ = 775 nm for both types, suggesting a cavity resonance of approximately *λ*_0_ ~ 1550 nm for the QWS mirrors.

### 3.1. Theoretical Predictions

The Purcell factor [11] is a useful place to start when analyzing the influence of the cavity on the electromagnetic mode characteristics and the cavity-emitter coupling. Many minutiae are accessible for analytical evaluation:

(1) $$\begin{array}{c} F_{P0}\equiv \frac{\gamma_{C}}{\gamma_{0}}=\frac{3}{4\pi^{2}}\frac{Q_{\text{EF}}}{\left(V_{M}/\lambda^{3}\right)}\;. \end{array}$$

Here, *γ*_*C*_ represents the emission rate in the cavity mode, *γ*_0_ is the emission rate in free space, *Q*_EF_ is the effective quality factor, and *V*_*M*_ is the mode volume. This expression presumes that the emitter is precisely aligned in space, spectrum, and polarization with the cavity mode [37], assuming the emitter is in air (*n*_air_ = 1). Furthermore, we can estimate Q_*EF*_ to be approximately equal to *Q*_*C*_, with *Q*_*C*_ representing the cavity quality factor for a small linewidth emitter, which is the focus of our consideration here. In the study [32], elliptical shapes were used to fabricate a concave elliptical upper mirror of a half-symmetric resonator. The upper mirrors exhibit distinct radii of curvature along each orthogonal axis of their ellipsoidal configuration structure. Assuming the values for radii curvature are 23.61 *μ* and 6.194 *μ*m and cavity height is 775 nm, the waist size [38] can be predicated according to *w*_*oj*_ = (*λ*_*o*_/*π*)^0.5^(*L*_EF_.*R*_*j*_)^0.25^ , where *j* for *x*, *y*, and *R*_*j*_ radii of curvature for *x*, *y*, and *L*_EF_ = *d*_*A*_ + 2*δ* is the effective length of the cavity combined with the penetration depth *δ*, which here is supposedly equal for the upper and lower mirrors. The penetration depth can be approximately represented [39] for mirrors with high index contrast as

(2) $$\begin{array}{c} \delta =\left(\frac{\lambda_{o}}{2}\right){\left(n_{H}-n_{L}\right)}^{-1}. \end{array}$$

Finally, the mode volume for fundamental-spatial-mode cavities can be represented by

(3) $$\begin{array}{c} V_{M}\approx \left(\frac{\pi }{4}\right)\cdot w_{0j}^{2}\cdot L_{\text{EF}}\;. \end{array}$$

Table 1 shows the predictions derived from the equations above for our cavity model.

## 4. Simulation Method Details

To simulate the spontaneous emission of our elliptical dome microcavity, we used a 3D numerical simulation (FDTD Lumerical software). The FDTD simulation of the plano elliptical shape was performed by incorporating the microcavity within a vacuum environment (refractive index, *n* = 1). In order to set boundary conditions for the FDTD simulation of the structure. A perfectly matched layer (PML) was added around the computational domain of the elliptical dome microcavity structure to absorb all incoming waves and prevent reflections from the boundaries. To enhance the coupling to the fundamental mode and reduce background emission, a dipole source has been placed exactly in the middle of the air core (embedded at the antinode of the electric field). Following Ref. [30], optical waves were produced, and high-order resonant modes were excited using a single broadband dipole source. We employed the FDTD software's built-in feature to compute the rate of the emission of an embedded dipole compared to its emission rate in free space (i.e., *γ*/*γ*_0_). Due to the dipole source being situated at the antinode of the fundamental cavity mode, the emission rate at this fundamental resonance frequency is roughly equivalent to the ideal Purcell factor *F*_*P*0_. On the other hand, the background radiation suppression can be represented by determining the off-resonance proportional to the emission rate. It is essential to ensure that the FDTD simulation runs long enough for the field values to diminish to a negligible level, allowing for an accurate estimation of the relative emission rates. Due to the relatively high photon lifetimes of the cavities, very long simulation times (up to ~1 week on a workstation laptop) were required and needed multirun to stabilize. In this work, we study two parts of simulation. Initially, we examine how the materials affect the cavity's optical characteristics, while in the second part, the study shows the impact of the ellipticity factor on the optical cavity.

### 4.1. Contrast Between TiO_2_- and ZnS-Based QWS Mirrors

**Figure 3:**
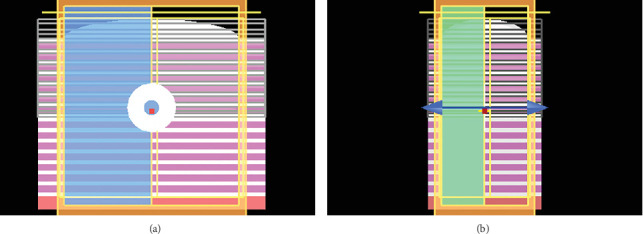
Modelled ellipsoid cavity of TiO_2_ and SiO_2_ as (TiO_2_/SiO_2_)^13^ side view: (a) YZ, (b) XZ.

The performance of QWS mirrors based on TiO_2_ and ZnS high-index layers is first compared. For the low-index layers in the two cases, we suppose SiO_2_.  Figure 3 shows two views of the device type studied here, which is TiO_2_-based, a plano ellipsoid shape with a semimajor and semiminor radii of 3 and 6 *μ*m, respectively, utilized in the first part of our simulation. Our devices were minimized in size to slightly reduce long simulations.  Figure 3a,b represents the modelled elliptical dome microcavity. The layers of material TiO_2_, material ZnS, and gap layer air are coloured white, pink, and black, respectively. The substrate material (silica wafer) with a refractive index of 1.46, used with elliptical dome microcavity, is coloured red. The dipole source of electromagnetic waves is represented by the blue spot with arrows, and the yellow line represents the monitors for measurement of electric field. The simulation domain is the orange box where all of the constructions are designed.

**Figure 4:**
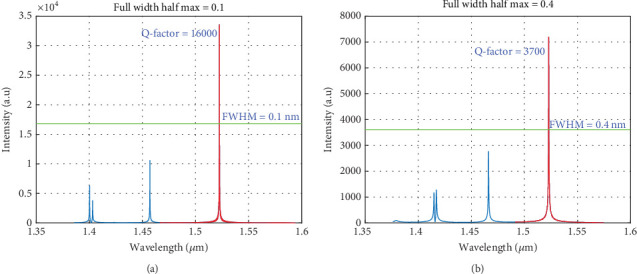
A transmission scan for a plano ellipsoid cavity. The *Q*-factor was estimated from the FWHM linewidth of (a) ~0.1 nm for the TiO_2_ cavity and (b) ~0.4 nm for the ZnS cavity.

**Figure 5:**
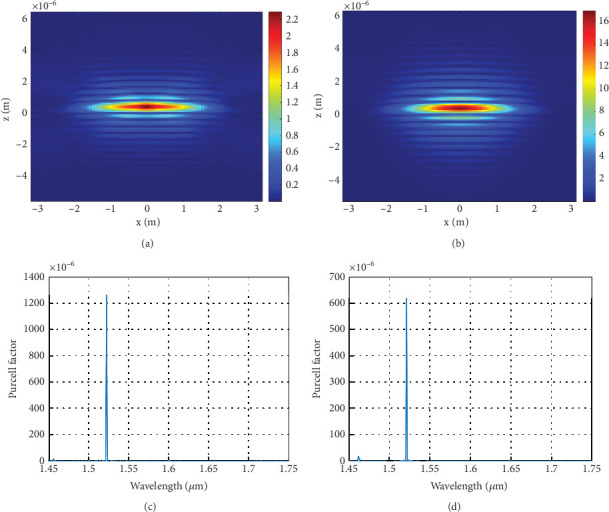
Cross-sectional mode field intensity profile of the fundamental mode for the (a) TiO_2_ ellipsoid cavity and the (b) ZnS ellipsoid cavity. (c) Relative dipole emission rate in relation to wavelength for the (d) TiO_2_ ellipsoid cavity, as in part but for the (c) ZnS ellipsoid cavity.

Figures 4 and 5 illustrate the main results from the FDTD simulations of the TiO_2_-based and ZnS-based cavities. From Figure 4, the TiO_2_-based mirror shows significant spatial confinement. The numerical calculations of the *Q*-factor values for the fundamental cavity resonance yielded results of approximately 16,000 for TiO_2_ and 3700 for ZnS, and they also agree well with the above predictions (slightly higher in the FDTD model because of the air superstrate). Additionally, the beam waists of the cavities were predicted to be around *w*_01_ 2.118 *μ*m (2.464 *μ*m) and *w*_02_ 1.299 *μ*m (1.577 *μ*m) (from the FDTD results) for TiO_2_ and ZnS, respectively; the predicted values are in good agreement with the numerical results.

Figure 5a,b shows the field intensity that is confined inside our devices (for an ellipse with cavity mirror separation of 775 nm, *d*_major_ = 6*  μ*m, and *d*_minor_ = 3*  μ*m). Comparing Figure 5a,b reveals more spatial confinement for the TiO_2_ case, with the analytically predicted values being in good agreement with the numerically predicted mode volumes of ∼2.5*λ*^3^ and ∼3.4*λ*^3^, respectively, while Figure 5c,d represents the relative emission versus the free space wavelength. From the plot, on- resonance values for both devices are ~1200 and 600, respectively. The FDTD results confirm that TiO_2_-based greatly reduce emission into background radiation modes and improved Purcell factor, mainly because of the smaller mode volume.

### 4.2. Effective Ellipticity Factor

**Figure 6:**
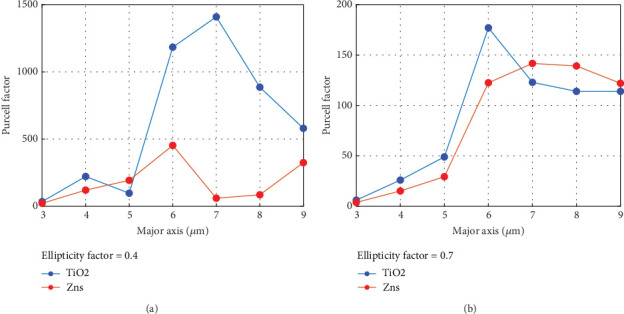
Purcell factor versus the major axis of the elliptical dome microcavity: (a) 0.4 ellipticity factor and (b) 0.7 ellipticity factor.

Using nominal values for the layer thicknesses and refractive indices, we simulated the plano ellipsoidal microcavity with various values for *d*_major_ and *d*_minor_. An in-plane dipole that is linearly polarized and positioned at the center of the mirror is utilized to excite the system. The Purcell factor was calculated by repeating the simulation for dipole alignment.  Figure 6 depicts the Purcell factor as a function of the length of the major axis of the elliptical microcavity plotted for two different plano elliptical cavities at a fixed value of ellipticity. Our numerical results revealed that the Purcell factor is susceptible to the plano elliptical microcavity size due to the lateral confinement of the photons in the cavity. As is easily recognized, ellipticity can significantly impact the Purcell factor, in which the TiO_2_-based cavity exhibits a higher Purcell factor compared to the ZnS-based cavity at ellipticity *ε* =0.4. Also, we observe a clear trend towards a higher Purcell factor for a larger ellipsoid cavity. The ellipticity, denoted as *ε*, can be calculated using the formula, $\varepsilon =\sqrt{a/b}-1$, in which *a* and *b* represent the lengths of the ellipse's semimajor and semiminor axes, respectively.

**Figure 7:**
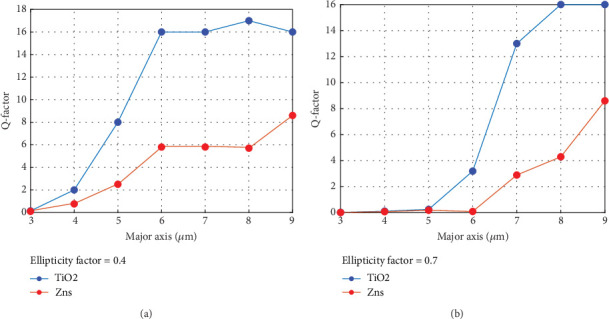
(a) The *Q*-factors are presented versus the major axis of the elliptical dome microcavities. A similar trend is observable in (b), which exhibits a greater ellipticity of the microcavities.

Subsequently, we investigated the quality factor of fundamental cavity modes in closely spaced elliptical dome microcavities.  Figure 7 illustrates how the *Q*-factors of the fundamental modes vary with the semimajor axis of our plano ellipsoid microcavities, plotted for two contrasting ellipticities: *ε* = 0.4 and *ε* = 0.7, respectively. It is evident that an increased extension of the plano ellipsoid microcavity results in a more excessive quality factor, attributable to diminished edge scattering losses, which consequently lead to reduced intrinsic losses. As can be seen in Figure 7, TiO_2_ exhibits a higher quality factor in comparison with ZnS, attributable to its reduced energy dissipation and enhanced dielectric properties.

**Figure 8:**
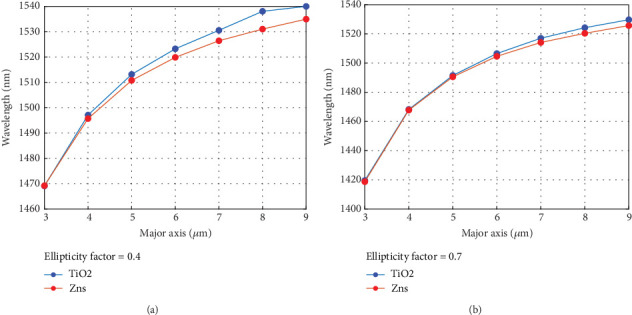
(a) The wavelengths are depicted versus the semimajor axis of the elliptical dome microcavities. A comparable trend is discernible in (b).

Figure 8 represents the fundamental wavelength of the plano elliptical cavity versus the fixed ellipticity and the semimajor axis of the elliptical microcavity. It is readily apparent that an increase in cavity dimensions results in a shift in wavelength (redshift) towards the longer range, caused by the decreasing effectiveness.

## 5. Discussion and Conclusion

The refractive index plays a key role in shaping the optical behavior of elliptical microcavities. The higher the refractive index, the stronger the light confinement, and the higher the spontaneous emission rate, and the more directional the radiation pattern. This makes high-refractive index materials ideal for photonic applications such as lasers, single-photon sources, and wavelength-selective emitters. However, optimizing device performance requires a careful balance of the trade-offs between absorption losses and fabrication complexity. The significant contrast in the refractive index of TiO_2_-based mirrors helps mitigate the increased film losses. Multilayer Bragg mirrors made from TiO_2_(ZnS)/SiO_2_ are seldom mentioned in the literature (refer to Refs. [40–42]). This might be linked to the difficulties of depositing high-quality TiO_2_(ZnS) and SiO_2_ films at one temperature during a single deposition run.

Our study shows that designing the elliptical dome cavity offers significant benefits for cavity QED applications, by increasing light–matter coupling (interaction between cavity mode and emitter) and providing control over spontaneous emission. As expected, an increase in ellipticity, accompanied by a decrease in area, results in a rise in the quality factor. Moreover, an expanded semimajor axis leads to a shift in wavelength towards the redshift. Numerical simulations show that emitters placed at strategic positions near the cavity focus increase the emission rate due to the concentration of the electric field in these regions. The Purcell factor increases with cavity eccentricity up to a critical value, beyond which the mode volume becomes too large, and the confinement quality deteriorates. In addition, by modulating the ellipticity factor, it is possible to optimize the fundamental wavelength for particular applications, including photonic crystal cavities and microring resonators. Elliptical cavities are frequently employed in the design of polarization-sensitive devices and for customizing mode behavior in optical filters and lasers. We believe that our study will serve as inspiration for more experiments in this field in the future.

## Figures and Tables

**Table 1:** Estimated parameters of the model cavity.

**Parameter**	**ZnS cavity**	**TiO**_**2**_ ** cavity**
QWS reflectance, *R*	0.999947 (*N* = 13)	0.999955 (*N* = 13)
QWS penetration, **δ**	968 (nm)	994 (nm)
Cavity *Q*, *Q*_*C*_ = *F*	2610	15707
Mode volume, *V*_*m*_	3*λ*_0_^3^	2*λ*_0_^3^
Mode radius, *w*_01_	1.996 (*μ*m)	1.9871 (*μ*m)
Mode radius, *w*_02_	1.4285 (*μ*m)	1.4221 (*μ*m)
Purcell factor, *F*_*P*0_	8 × 10^3^	16 × 10^3^
Index, *N*_*H*_	2.25 − *i*0.0000011	2.45 − *i*9.3247e − 284

## Data Availability

The data used to support the findings of this study are available from the corresponding author upon request.
